# Nonlinear, Nonhomogeneous Periodic Problems with no Growth Control on the Reaction

**DOI:** 10.1007/s10883-014-9245-4

**Published:** 2014-08-30

**Authors:** Leszek Gasiński, Nikolaos S. Papageorgiou

**Affiliations:** 1 Faculty of Mathematics and Computer Science, Jagiellonian University, ul. Łojasiewicza 6, 30-348 Kraków, Poland; 2 Department of Mathematics, National Technical University, Zografou Campus, 157 80 Athens, Greece

**Keywords:** Nonhomogeneous differential operator, Nonlinear strong maximum principle, Constant sign solutions, Nodal solutions, Mountain pass theorem, Second deformation theorem, 34B15, 34B18

## Abstract

We consider a nonlinear periodic problem driven by a nonhomogeneous differential operator, which includes as a particular case the scalar *p*-Laplacian. We assume that the reaction is a Carathéodory function which admits time-dependent zeros of constant sign. No growth control near ±∞ is imposed on the reaction. Using variational methods coupled with suitable truncation and comparison techniques, we prove two multiplicity theorems providing sign information for all the solutions.

## Introduction

In this paper, we study the following nonlinear periodic problem:

1.1 $$ \left\{ \begin{array}{l} -\left(a\left(|u^{\prime}(t)|\right)u^{\prime}(t)\right)'\ =\ f\left(t,u(t)\right)\quad\textrm{a.e. on}\ T=[0,b],\\ u(0)=u(b),\ u^{\prime}(0)=u^{\prime}(b). \end{array} \right. $$

Here, the differential operator is in general nonhomogeneous and incorporates as special cases the scalar *p*-Laplacian, the scalar (*p*,*q*)-Laplacian and the scalar generalized *p*-mean curvature differential operator. The reaction *f*(*t*,*ζ*) is a Carathéodory function (i.e. for all ζ ∈ ℝ, the function *t* ↦ *f*(*t*,*ζ*) is measurable, and for almost all *t*∈*T*, the function *ζ*↦*f*(*t*,*ζ*) is continuous) which has constant sign, *t*-dependent zeros. Our aim is to prove multiplicity theorems for problem (1.1), providing precise sign information for all the solutions.

In fact, our conditions on the reaction *f*(*t*,*ζ*) are simple and easy to verify and incorporate into our framework several interesting applied cases. Essentially, we require that the reaction *f*(*t*,⋅) exhibits a kind of oscillatory behaviour near zero. For example, consider the following semilinear periodic problem:

$$\left\{ \begin{array}{l} -u^{\prime\prime}\ =\ \alpha u-\beta u^{2}+\gamma u^{3},\\ u(0)=u(b),\ u^{\prime}(0)=u^{\prime}(b), \end{array} \right. $$

with *α*,*β*,*γ*>0 such that *β* ^2^−4*α* *γ*>0. For this problem, the reaction is autonomous (*t*-independent) and has the form

$$f(\zeta) \ =\ \zeta \left(\alpha-\beta\zeta+\gamma\zeta^{2}\right). $$

Since $\frac {\beta \pm \sqrt {\beta ^{2}-4\alpha \gamma }}{2\gamma }>0$, there exist 0<*ζ*_0_<*ζ*_1_ such that *f*(*ζ*_0_)=*f*(*ζ*_1_)=0. Then according to Proposition 3.4, this problem has a positive solution. This equation is a homogeneous version of a problem studied by Cronin-Scanlon [8] in the context of a biomathematical model of aneurysm. In fact, we can add in the reaction a suitable perturbation *h*(*ζ*) with no growth restriction, provided that it has suitable oscillatory behaviour near zero.

Our framework also incorporates logistic equations of the following form:

$$\left\{ \begin{array}{l} -u^{\prime\prime}\ =\ u-u^{q-1},\ u>0,\\ u(0)=u(b),\ u^{\prime}(0)=u^{\prime}(b), \end{array} \right. $$

with *q* > 2. In this case, *f*(*ζ*)=*ζ*−*ζ* ^*q*−1^=*ζ*(1−*ζ* ^*q*−2^), *ζ*>0, and we infer that the problem has a positive solution. Of course we can have a reaction of the form *f*(*ζ*)=*ζ*−|*ζ*|^*q*−2^ *ζ, ζ* ∈ ℝ, and then we can guarantee also negative solutions (see Proposition 3.4). We may include harvesting, that is

$$f(\zeta)\ =\ \zeta-\zeta^{q-1}h(\zeta),\quad\text{with}\ h>0. $$

Usually the harvesting is proportional to the population, that is *h*(*ζ*)=*c* *ζ*, *c*>0. Then

$$f(\zeta)\ =\ (1-c)\zeta-\zeta^{q-1},\quad \zeta>0. $$

If *c*∈(0,1), then we are back to the previous situation. In fact for such problems, the function $\zeta \longmapsto \frac {f(\zeta )}{\zeta }$ is strictly decreasing on $(0,+\infty )$ and so according to Kyritsi-Papageorgiou [19], the positive solution is unique.

Other possibility is a reaction of the form

$$f(\zeta)\ =\ \zeta^{q-1}-\zeta,\quad \zeta>0, $$

with *q*>2, which arises in chemotaxis models.

The reaction

$$f(\zeta)\ = \ |\zeta|^{\tau-2}\zeta-|\zeta|^{q-2}\zeta,\quad\zeta\in\mathbb{R}, $$

with *τ*<*p*<*q*, leads to a logistic-type equation of subdiffusive type and fits in the framework of Theorem 3.11. So, the corresponding equation driven by the scalar *p*-Laplacian has at least three solutions, two of constant sign and the third nodal.

Thus, we see that our setting is general and rather natural in the context of many applied problems.

In this paper, we prove two “three-solution theorems,” in which we produce a positive, a negative and a nodal (sign changing) solutions. The assumption that *f*(*t*,⋅) has zeros implies that we do not need to impose any growth control near ±∞ for the function (*t*,⋅). Our approach is variational based on the critical point theory, coupled with suitable truncation and comparison techniques.

Multiplicity results for the periodic scalar *p*-Laplacian were proved by Aizicovici-Papageorgiou-Staicu [1, 5, 6], del Pino-Manásevich-Murúa [9], Gasiński [11], Gasiński-Papageorgiou [15–17] and Zhang-Liu [24]. None of the aforementioned works produce nodal solutions. For periodic problems with more general operators, we refer to Gasiński [12] and Gasiński-Papageorgiou [18].

In the next section, for the convenience of the reader, we present the main mathematical tools which we will use in this work.

## Mathematical Background - Hypotheses

Let *X* be a Banach space and let *X* ^∗^ be its topological dual. By 〈⋅,⋅〉, we denote the duality brackets for the pair (*X*,*X* ^∗^). We say that *φ*∈*C* ^1^(*X*) satisfies the *Palais-Smale condition* if the following is true:

“Every sequence $\{x_{n}\}_{n\geqslant 1}\subseteq X$, such that $\left \{\varphi (x_{n})\right \}_{n\geqslant 1}\subseteq \mathbb {R}$ is bounded and

$$\varphi^{\prime}(x_{n})\ \longrightarrow\ 0 \quad\text{in}\ X^{*}, $$

admits a strongly convergent subsequence.”

Using this compactness-type condition, we can prove the following minimax theorem, known in the literature as the “mountain pass theorem.”

### **Theorem 2.1**

*If X is a Banach space, φ∈C* ^1^ *(X) satisfies the Palais-Smale condition, x*_0_ *,x*_1_ *∈X, ∥x*_1_ *−x*_0_ *∥>r>0,*

$$\max\left\{\varphi(x_{0}),\varphi(x_{1})\right\} \ <\ \inf\left\{\varphi(x):\ \|x-x_{0}\|=r\right\} \ =\ \eta_{r}, $$

*and*

$$c \ =\ \inf_{\gamma\in{\Gamma}}\max_{t\in [0,1]}\varphi\left(\gamma(t)\right), $$

*where*

$${\Gamma} \ =\ \left\{\gamma\in C\left([0,1];X\right):\ \gamma(0)=x_{0},\ \gamma(1)=x_{1}\right\}, $$

*then* $c\geqslant \eta _{r}$ *and c is a critical value of φ.*

Another result from critical point theory which we will need in the sequel is the so-called second deformation theorem (see, e.g. Gasiński-Papageorgiou [13, p. 628]). Let *φ*∈*C* ^1^(*X*) and let $c\in \mathbb {R}$. We introduce the following sets:

$$\begin{array}{@{}rcl@{}} \varphi^{c} & = &\left\{ x\in X:\ \varphi(x)\leqslant c\right\},\\ K_{\varphi} & = &\left\{x\in X:\ \varphi^{\prime}(x)=0\right\},\\ K_{\varphi}^{c} & = &\left\{x\in K_{\varphi}:\ \varphi(x)=c\right\}. \end{array} $$

### **Theorem 2.2**

*If φ∈C* ^1^ *(X),* $a\in \mathbb {R}$ *,* $a<b\leqslant +\infty $ *, φ satisfies the Palais-Smale condition, φ has no critical values in (a,b) and φ* ^*−1*^ *({a}) contains at most a finite number of critical points of φ, then there exists a homotopy* $h\colon [0,1]\times (\varphi ^{b}\setminus K_{\varphi }^{b})\longrightarrow \varphi ^{b}$ *, such that*

**(a)** *h* (1, *φ* ^*b*^ \ *K*_φ_^*b*^) ⊆ *φ* ^*a*^;

**(b)** *h*(*t, x*) = *x for all t* ∈ [0, 1], *all x* ∈ *φ* ^*a*^;

**(c)** *φ* (*h*(*t, x*)) ≤ *φ* (*h*(*s, x*)) *for all t, s* ∈ [0, 1], *s* ≤ *t, all x* ∈ *X*.

### *Remark 2.3*

In particular, Theorem 2.2 implies that *φ* ^*a*^ is a strong deformation retract of $\varphi ^{b}\setminus K_{\varphi }^{b}$. Hence, the two sets are homotopy equivalent.

In the study of problem (1.1), we will use the following two spaces:

$$\begin{array}{@{}rcl@{}} W^{1,p}_{\text{per}}(0,b) & = &\left\{u\in W^{1,p}(0,b):\ u(0)=u(b)\right\},\\ \widehat{C}^{1}(T) & = & C^{1}(T)\cap W^{1,p}_{\text{per}}(0,b), \end{array} $$

where $1<p<+\infty $. Recall that the Sobolev space *W* ^1,*p*^(0,*b*) is embedded continuously (in fact compactly) in *C*(*T*), and so the evaluations at *t*=0 and *t*=*b* of *u*∈*W* ^1,*p*^(0,*b*) make sense. The Banach space $\widehat {C}^{1}(T)$ is an ordered Banach space with a positive cone

$$\widehat{C}_{+} \ =\ \left\{u\in \widehat{C}^{1}(T):\ u(t)\geqslant 0\ \textrm{for all}\ t\in T\right\}. $$

This cone has a nonempty interior given by

$$\text{int}\, \widehat{C}_{+} \ =\ \left\{u\in\widehat{C}_{+}:\ u(t)>0\ \textrm{for all}\ t\in T\right\}. $$

Consider the following nonlinear eigenvalue problem:

2.1 $$ \left\{ \begin{array}{l} -\left(|u^{\prime}(t)|^{p-2}u^{\prime}(t)\right)'\ =\ \lambda |u(t)|^{p-2}u(t)\quad\textrm{a.e. on}\ T=[0,b],\\ u(0)=u(b),\ u^{\prime}(0)=u^{\prime}(b), \end{array} \right. $$

where 1 < *p* < + ∞. A number *λ* ∈ ℝ is said to be an *eigenvalue* of the negative periodic scalar *p*-Laplacian if problem (2.1) has a nontrivial solution, which is a corresponding *eigenfunction*. Evidently, a necessary condition for *λ* ∈ ℝ to be an eigenvalue is that *λ* ≥ 0. We see that *λ*_0_=0 is an eigenvalue and the corresponding eigenfunctions are constant functions (i.e. the corresponding eigenspace is ℝ). Let

$$\pi_{p} \ =\ \frac{2\pi (p-1)^{\frac{1}{p}}}{p\sin \frac{\pi}{p}}. $$

Then $\left \{\lambda _{n}=\left (\frac {2n\pi _{p}}{b}\right )^{p}\right \}_{n\geqslant 0}$ is the set of eigenvalues of Eq. 2.1. If *p*=2 (linear eigenvalue problem), then *π*_2_=*π* and so we recover the well-known sequence of eigenvalues of the negative periodic scalar Laplacian, which is $\left \{\lambda _{n}=\left (\frac {2n\pi }{b}\right )^{2}\right \}_{n\geqslant 0}$. Every eigenfunction *u*∈*C* ^1^(*T*) of Eq. 2.1 satisfies

$$u(t)\ \ne\ 0\quad\textrm{a.e. on}\ T $$

(in fact, it has a finite number of zeros) and all eigenfunctions corresponding to an eigenvalue *λ*>*λ*_0_=0 are nodal (see Aizicovici-Papageorgiou-Staicu [3]).

Let $\widehat {u}_{0}$ be the *L* ^*p*^-normalized principal (i.e. corresponding to *λ*_0_=0) eigenfunction. Hence,

$$\widehat{u}_{0}(t) \ =\ \frac{1}{b^{\frac{1}{p}}} \quad\forall t\in T. $$

Also, let

$$\begin{array}{@{}rcl@{}} \partial B_{1}^{L^{p}} & = &\left\{u\in L^{p}(T):\ \|u\|_{p}=1\right\},\\ M & = & W^{1,p}_{\text{per}}(0,b)\cap \partial B_{1}^{L^{p}}. \end{array} $$

For *λ*_1_>0 (the first nonzero eigenvalue), we have the following variational characterization (see Aizicovici-Papageorgiou-Staicu [4, 5]).

### **Proposition 2.4**

*If*

$$\widehat{\Gamma}=\left\{\widehat{\gamma}\in C\left([-1,1];M\right):\ \widehat{\gamma}(-1)=-\widehat{u}_{0}, \ \widehat{\gamma}(1)=\widehat{u}_{0}\right\}, $$

*then*

$$\lambda_{1} \ =\ \inf_{\widehat{\gamma}\in\widehat{\gamma}}\max_{-1\leqslant s\leqslant 1} \big\|\frac{d}{dt}\widehat{\gamma}(s)\big\|_{p}^{p}. $$

The hypotheses on the map *a* are the following: *H(a)* *a* : (0, + ∞) → (0, + ∞) is a *C* ^1^-function, such that

(i)  the function *ζ* ↦ *a*(*ζ*)*ζ* is strictly increasing on (0, +∞) and

$$\lim_{\zeta\rightarrow 0^{+}}a(\zeta)\zeta\ =\ 0,\quad\text{and}\quad \lim_{\zeta\rightarrow 0^{+}}\frac{a^{\prime}(\zeta)\zeta}{a(\zeta)}\ =\ c\ >\ -1; $$

(ii)  there exist *c*_0_>0 and *p* ∈ (1, + ∞), such that

$$c_{0}|\zeta|^{p-1}\ \leqslant\ a^{\prime}(|\zeta|)\zeta^{2} \quad\forall \zeta\in\mathbb{R}; $$

(iii)  there exists *c*_1_>0 such that

$$\big| a(|\zeta|)\zeta\big|\ \leqslant\ c_{1}\left(1+|\zeta|^{p-1}\right) \quad\forall \zeta\in\mathbb{R}; $$

(iv)  if

$$G_{0}(t) \ =\ {{\int}_{0}^{t}} a(s)s\,ds\quad\forall t>0, $$

then

$$p G_{0}(\zeta)-a(\zeta)\zeta^{2}\ \geqslant\ -\widetilde{c} \quad\forall \zeta\geqslant 0, $$

with $\widetilde {c}>0$ and there exists *τ*∈(1,*p*) such that

$$\lim_{t\rightarrow 0^{+}}\frac{G_{0}(t)}{t^{\tau}}\ =\ 0. $$

### *Remark 2.5*

Evidently, the function *G*_0_: [0, + ∞) → [0, + ∞) introduced in hypothesis *H*(*a*)(iv) is strictly convex and strictly increasing. We set

$$G(\zeta) \ =\ G_{0}(|\zeta|) \quad\forall \zeta\in\mathbb{R}. $$

Then *G*(0)=0 and we have

$$G^{\prime}(\zeta) \ =\ G_{0}^{\prime}(|\zeta|)\frac{\zeta}{|\zeta|} \ =\ a(|\zeta|)\zeta \quad\forall \zeta\ne 0, $$

while $G^{\prime }(0)=0$ (see hypothesis *H*(*a*)(i)). Therefore, the function *ζ*↦*G*(*ζ*) is the primitive of the function *ζ*↦*a*(|*ζ*|)*ζ*. Then *G* is strictly convex and so

2.2 $$ G(\zeta)\ \leqslant\ a(|\zeta|)\zeta^{2} \quad\forall \zeta\in\mathbb{R}. $$

Then from hypotheses *H*(*a*) and Eq. 2.2, we obtain

2.3 $$ \frac{c_{0}}{p}|\zeta|^{p} \ \leqslant\ G(\zeta) \ \leqslant\ c_{2}\left(1+|\zeta|^{2}\right) \quad\forall \zeta\in\mathbb{R}, $$

for some *c*_2_>0.

### *Example 2.6*

The following functions *a*(⋅) satisfy hypotheses *H*(*a*):

**(a)**  *a*(|*ζ*|)*ζ*=|*ζ*|^*p*−2^ *ζ*, with 1 < *p* < + ∞. This map corresponds to the scalar *p*-Laplacian.

**(b)**  *a*(|*ζ*|)*ζ*=|*ζ*|^*p*−2^ *ζ*+|*ζ*|^*q*−2^ *ζ*, with 1 < *q* < *p* < + ∞. This map corresponds to the (*p*,*q*)-Laplace differential operator (the sum of a scalar *p*-Laplacian with a scalar *q*-Laplacian).

**(c)**  $a(|\zeta |)\zeta =\left (1+\zeta ^{2}\right )^{\frac {p-2}{2}}\zeta $, with 1 < *p* < +∞. This map corresponds to the scalar generalized *p*-mean curvature operator.

**(d)**  $a(|\zeta |)=|\zeta |^{p-2}\zeta +\frac {|\zeta |^{p-2}\zeta }{1+|\zeta |^{p}}$ with 1 < *p* < +∞.

In what follows, for notational economy, we write $W=W^{1,p}_{\text {per}}(0,b)$. We introduce the nonlinear map *A*:*W*→*W* ^∗^, defined by

2.4 $$ \left\langle A(u),y\right\rangle \ =\ {{\int}_{0}^{b}} a\left(|u^{\prime}(t)|\right)u^{\prime}(t)y^{\prime}(t)\,dt \quad\forall u,y\in W. $$

From Papageorgiou-Rocha-Staicu [21], we have the following result concerning the map *A*.

### **Proposition 2.7**

*If hypotheses H(a) hold, then A:W→W* ^*∗*^ *defined by Eq.* 2.4 *is continuous, bounded (i.e. maps bounded sets to bounded ones), maximal monotone and of type (S)*_*+*_ *, i.e. if u*_*n*_ *→wu in W and* $\limsup \limits _{n\rightarrow +\infty }\left \langle A(u_{n}),u_{n}-u\right \rangle \leqslant 0$ *, then u*_*n*_ *→u in W.*

Let *f*_0_ : *T* × ℝ → ℝ be a Carathéodory function, such that

$$\big|f_{0}(t,x)\big| \ \leqslant\ \vartheta(t)\left(1+|\zeta|^{r-1}\right) \quad\textrm{for almost all}\ t\in T,\ \text{all}\ \zeta\in\mathbb{R}, $$

with *𝜗*∈*L* ^1^(*T*)_+_, 1 < *r* < +∞. We set

$$F_{0}(t,\zeta) \ =\ {\int}_{0}^{\zeta} f_{0}(t,s)\,ds $$

and consider the *C* ^1^-functional *σ*_0_: *W* → ℝ, defined by

$$\sigma_{0}(u) \ =\ {{\int}_{0}^{b}} G\left(u^{\prime}(t)\right)\,dt -{{\int}_{0}^{b}} F_{0}\left(t,u(t)\right)\,dt \quad\forall u\in W. $$

Then as in Aizicovici-Papageorgiou-Staicu [1] (see Proposition 9, where $G(\zeta )=\frac {1}{p}|\zeta |^{p}$), we can have the following result relating local $\widehat {C}^{1}(T)$-minimizers and local *W*-minimizers for the functional *σ*_0_ (cf. also Gasiński-Papageorgiou [18, Proposition 2.5]).

### **Proposition 2.8**

*If hypotheses H(a) hold and u*_0_ *∈W is a local* $\widehat {C}^{1}(T)$ *-minimizer of σ*_0_ *, i.e. there exists ϱ*_0_ *>0, such that*

$$\sigma_{0}(u_{0})\ \leqslant\ \sigma_{0}(u_{0}+h) \quad\forall h\in \widehat{C}^{1}(T),\ \text{with}\ \|h\|_{\widehat{C}^{1}(T)}\leqslant \varrho_{0}, $$

*then* $u_{0}\in \widehat {C}^{1}(T)$ *and it is also a local W-minimizer of σ*_0_ *, i.e. there exists ϱ*_1_ *>0, such that*

$$\sigma_{0}(u_{0})\ \leqslant\ \sigma_{0}(u_{0}+h) \quad\forall h\in W,\ \text{with}\ \|h\|\leqslant \varrho_{1}. $$

Throughout this paper, by ∥⋅∥, we denote the norm of the Sobolev space $W=W^{1,p}_{\text {per}}(0,b)$. The norm of *L* ^*p*^(*T*) ($1\leqslant p\leqslant +\infty $) is denoted by ∥⋅∥_*p*_, while by →*w*, we denote the weak convergence in any Banach space. If $\zeta \in \mathbb {R}$, then we set

$$\zeta^{+}\ =\ \max\{\zeta,0\} \quad\text{and}\quad \zeta^{-}\ =\ \max\{-\zeta,0\}. $$

We have *ζ*=*ζ* ^+^−*ζ* ^−^ and |*ζ*|=*ζ* ^+^+*ζ* ^−^. If *u*∈*W*, we define

$$u^{+}(\cdot)\ =\ u(\cdot)^{+} \quad\text{and}\quad u^{-}(\cdot)\ =\ u(\cdot)^{-}. $$

We know that *u* ^+^,*u* ^−^∈*W* and *u*=*u* ^+^−*u* ^−^, |*u*|=*u* ^+^+*u* ^−^. By |⋅|_1_ we denote the Lebesgue measure on $\mathbb {R}$ and if $h\colon T\times \mathbb {R}\longrightarrow \mathbb {R}$ is a measurable function (for example, a Carathéodory function), then we set

$$N_{h}(u)(\cdot) \ =\ h\left(\cdot,u(\cdot)\right) \quad\forall u\in W. $$

## Three Solution Theorems

In this section, we prove two multiplicity theorems for problem (1.1) providing sign information for all the solutions.

To produce the constant sign solutions, we will need the following hypotheses on the reaction *f*:

*H(f)*_1_ *f* : *T* × ℝ → ℝ is a Carathéodory function, such that *f*(*t*,0)=0 for almost all *t*∈*T* and

(i)  for every *ϱ*>0, there exists *a*_*ϱ*_∈*L* ^1^(*T*)_+_, such that

$$\big|f(t,\zeta)\big|\ \leqslant\ a_{\varrho}(t) \quad\textrm{for almost all}\ t\in T,\ \text{all}\ |\zeta|\leqslant\varrho; $$

(ii)  there exist functions *w*_±_∈*W*, such that

$$w_{-}(t)\ \leqslant\ c_{-}\ <\ 0\ <\ c_{+}\ \leqslant\ w_{+}(t) \quad\forall t\in T, $$

$$f\left(t,w_{+}(t)\right)\ \leqslant\ 0\ \leqslant\ f\left(t,w_{-}(t)\right) \quad\textrm{for a.a.}\ t\in T $$

and

$$A(w_{-})\ \leqslant\ 0\ \leqslant\ A(w_{+})\quad\text{in}\ W^{*}; $$

(iii)  there exist $\delta _{0}\in \left (0,\min \{c_{+},-c_{-}\}\right )$ and $T_{0}\subseteq T$ with |*T*_0_|_1_>0, such that

$$\begin{array}{@{}rcl@{}} \left\{ \begin{array}{ll} f(t,\zeta)\zeta\geqslant 0 & \textrm{for almost all}\ t\in T,\ \text{all}\ |\zeta|\leqslant\delta_{0},\\ f(t,\zeta)\zeta>0 & \textrm{for almost all}\ t\in T_{0},\ \text{all}\ 0<|\zeta|\leqslant\delta_{0}; \end{array} \right. \end{array} $$

(iv)  there exists *ξ*_∗_>0, such that

$$f(t,\zeta)\zeta+\xi_{*}|\zeta|^{p}\ \geqslant\ 0 \quad\textrm{for almost all}\ t\in T,\ \text{all}\ \zeta\in [-m_{*},m_{*}], $$

where $m_{*}=\max \left \{\|w_{+}\|_{\infty },\|w_{-}\|_{\infty }\right \}$.

### *Remark 3.1*

Hypotheses *H*(*f*)_1_(ii) and (iii) imply that for almost all *t*∈*T*, *f*(*t*,⋅) has *t*-dependent zeros of constant sign. The presence of these zeros frees *f*(*t*,⋅) from any growth restrictions near ±∞. Note that we do not impose any control on the growth of *f*(*t*,⋅) near ±∞. Hypothesis *H*(*f*)_1_(ii) is satisfied if we can find *c*_−_< 0 <*c*_+_, such that

$$f(t,c_{+})\ \leqslant\ 0\ \leqslant\ f(t,c_{-}) \quad\textrm{for almost all}\ t\in T. $$

We start by showing that the nontrivial constant sign solutions of Eq. 1.1 have *L* ^∞^ norms which are bounded away from zero.

### **Proposition 3.2**

*If hypotheses H(a) and H(f)*_1_ *hold and* $u\in \widehat {C}_{+}\setminus \{0\}$ *,* $v\in (-\widehat {C}_{+})\setminus \{0\}$ *are solutions of Eq.* 1.1 *, then* $\delta _{0}\leqslant \|u\|_{\infty }$ *and* $\delta _{0}\leqslant \|v\|_{\infty }$ *, where δ*_0_ *>0 is as in hypothesis H(f)*_1_ *(iii).*

### *Proof*

Since by hypothesis $u\in \widehat {C}_{+}\setminus \{0\}$ is a solution of Eq. 1.1, we have

3.1 $$ A(u) \ =\ N_{f}(u). $$

Suppose that $\|u\|_{\infty }<\delta _{0}$. Acting on Eq. 3.1 with $h\equiv 1\in \widehat {C}_{+}$, we obtain

$$0 \ =\ {{\int}_{0}^{b}} f\left(t,u(t)\right)\,dt $$

(see Eq. 2.4), so

$$f\left(t,u(t)\right) \ =\ 0 \quad\textrm{for almost all}\ t\in T $$

(since $0\leqslant u(t)<\delta _{0}$ for all *t*∈*T*; see hypothesis *H*(*f*)_1_(iii)). This contradicts hypothesis *H*(*f*)_1_(iii). Therefore, $\|u\|_{\infty }\geqslant \delta _{0}$. □

Similarly for *v* ∈ (−*Ĉ*_+_) ∖ {0}. □

Next, we establish the existence of nontrivial solutions of constant sign.

### **Proposition 3.3**

*If hypotheses H(a) and H(f) hold, then problem (* 1.1 *) has at least one nontrivial positive solution* $u_{0}\in \text {int}\, \widehat {C}_{+}$ *and at least one nontrivial negative solution* $v_{0}\in -\text {int}\, \widehat {C}_{+}$.

### *Proof*

First, we produce the nontrivial positive solution. To this end, we consider the following truncation-perturbation of the reaction *f*:

3.2 $$ \widehat{f}_{+}(t,\zeta) \ =\ \left\{ \begin{array}{lll} 0 & \text{if} & \zeta<0,\\ f(t,\zeta)+\zeta^{p-1} & \text{if} & 0\leqslant \zeta\leqslant w_{+}(t),\\ f(t,w_{+}(t))+w_{+}(t)^{p-1} & \text{if} & w_{+}(t)<\zeta. \end{array} \right. $$

This is a Carathéodory function. Let

$$\widehat{F}_{+}(t,\zeta) \ =\ {\int}_{0}^{\zeta}\widehat{f}_{+}(t,s)\,ds $$

and consider the *C* ^1^-functional $\widehat {\varphi }_{+}\colon W\longrightarrow \mathbb {R}$, defined by

$$\widehat{\varphi}_{+}(u) \ =\ {{\int}_{0}^{b}} G\left(u^{\prime}(t)\right)\,dt +\frac{1}{p}\|u\|_{p}^{p} -{{\int}_{0}^{b}} \widehat{F}_{+}\left(t,u(t)\right)\,dt \quad\forall u\in W. $$

It is clear from Eq. 2.3 and Eq. 3.2 that $\widehat {\varphi }_{+}$ is coercive. Also, using the Sobolev embedding theorem, we see that $\widehat {\varphi }_{+}$ is sequentially weakly lower semicontinuous. So, by virtue of the Weierstrass theorem, we can find *u*_0_∈*W*, such that

3.3 $$ \widehat{\varphi}_{+}(u_{0}) \ =\ \inf_{u\in W}\widehat{\varphi}_{+}(u) \ =\ \widehat{m}_{+}. $$

Let *ξ*∈(0,*δ*_0_]. Then, for

$$F(t,\zeta) \ =\ {\int}_{0}^{\zeta}f(t,s)\,ds, $$

we have

$$\widehat{\varphi}_{+}(\xi) \ =\ -{{\int}_{0}^{b}} F(t,\xi)\,dt \ <\ 0 $$

(see Eq. 3.2 and hypothesis *H*(*f*)_1_(iii)), so

$$\widehat{\varphi}_{+}(u_{0}) \ =\ \widehat{m}_{+} \ <\ 0 \ =\ \widehat{\varphi}_{+}(0), $$

i.e. *u*_0_≠0. From Eq. 3.3, we have

$$\widehat{\varphi}_{+}^{\prime}(u_{0}) \ =\ 0, $$

so

3.4 $$ A(u_{0})+|u_{0}|^{p-2}u_{0} \ =\ N_{\widehat{f}_{+}}(u_{0}). $$

Acting on Eq. 3.4 with $-u_{0}^{-}\in W$, we obtain

$$c_{0}\left\|(u_{0}^{-})^{\prime}\right\|_{p}^{p} +\|u_{0}^{-}\|_{p}^{p} \ \leqslant\ 0 $$

(see hypothesis *H*(*a*)(ii) and Eq. 3.2), so

$$u_{0}^{-}\ =\ 0, $$

hence $u_{0}\geqslant 0$, *u*_0_≠0. Then from Eq. 3.4 and Eq. 3.2, we have

$$A(u_{0})\ =\ N_{f}(u_{0}), $$

so

$$\left\{ \begin{array}{l} -\left(a\left(|u_{0}^{\prime}(t)|\right)u_{0}^{\prime}(t)\right)'\ =\ f\left(t,u_{0}(t)\right)\quad\textrm{a.e. on}\ T,\\ u_{0}(0)=u_{0}(b),\ u_{0}^{\prime}(0)=u_{0}^{\prime}(b), \end{array} \right. $$

so $u_{0}\in \widehat {C}_{+}\setminus \{0\}$ solves problem (1.1).

Moreover, hypothesis *H*(*f*)_1_(iv) implies that

$$a\left(|u_{0}^{\prime}(t)|\right)u_{0}^{\prime}(t) \ \leqslant\ \xi_{*} u_{0}(t)^{p-1} \quad\textrm{almost everywhere on}\ T, $$

so $u_{0}\in \text {int}\, \widehat {C}_{+}$ (see Pucci-Serrin [22, p. 120])

For the nontrivial negative solution, we consider

$$\widehat{f}_{-}(t,\zeta) \ =\ \left\{ \begin{array}{lll} f(t,w_{-}(t))+|w_{-}(t)|^{p-2}w_{-}(t) & \text{if} & \zeta<w_{+}(t),\\ f(t,\zeta)+|\zeta|^{p-2}\zeta & \text{if} & w_{-}(t)\leqslant\zeta\leqslant 0,\\ 0 & \text{if} & 0<\zeta. \end{array} \right. $$

This is a Carathéodory function. We set

$$\widehat{F}_{-}(t,\zeta) \ =\ {\int}_{0}^{\zeta}\widehat{f}_{-}(t,s)\,ds $$

and consider the *C* ^1^-functional $\widehat {\varphi }_{-}\colon W\longrightarrow \mathbb {R}$, defined by

$$\widehat{\varphi}_{-}(u) \ =\ {{\int}_{0}^{b}} G\left(u^{\prime}(t)\right)\,dt +\frac{1}{p}\|u\|_{p}^{p} -{{\int}_{0}^{b}} \widehat{F}_{-}\left(t,u(t)\right)\,dt \quad\forall u\in W. $$

Reasoning as above, via the direct method, we obtain a nontrivial negative solution $v_{0}\in -\text {int}\, \widehat {C}_{+}$. □

In fact, we can show that (1.1) admits extremal nontrivial constant sign solution, i.e. there is the smallest nontrivial positive solution and biggest nontrivial negative solution.

### **Proposition 3.4**

*If hypotheses H(a) and H(f) hold, then problem (* 1.1 *) has the smallest nontrivial positive solution* $u_{*}\in \text {int}\, \widehat {C}_{+}$ *and biggest nontrivial negative solution* $v_{*}\in -\text {int}\, \widehat {C}_{+}$.

### *Proof*

First, we show the existence of the smallest nontrivial positive solution. Let *ξ*∈(0,*δ*_0_] (where *δ*_0_>0 is as in hypothesis *H*(*f*)_1_(iii)) and consider the order interval

$$[\xi,w_{+}]~=~\left\{u\in W:\ \xi\leqslant u(t)\leqslant w_{+}(t)\ \textrm{for almost all}\ t\in T\right\}. $$

*Claim 1.* Problem (1.1) has a solution in the order interval [*ξ*,*w*_+_].

To this end, we consider the following truncation-perturbation of *f*(*t*,⋅):

3.5 $$ k_{+}(t,\zeta) \ =\ \left\{ \begin{array}{lll} f(t,\xi)+\xi^{p-1} & \text{if} & \zeta<\xi,\\ f(t,\zeta)+\zeta^{p-1} & \text{if} & \xi\leqslant \zeta\leqslant w_{+}(t),\\ f(t,w_{+}(t))+w_{+}(t)^{p-1} & \text{if} & w_{+}(t)<\zeta. \end{array} \right. $$

This is a Carathéodory function. Let

$$K_{+}(t,\zeta) \ =\ {\int}_{0}^{\zeta}k_{+}(t,s)\,ds $$

and consider the *C* ^1^-functional $\psi _{+}\colon W\longrightarrow \mathbb {R}$, defined by

$$\psi_{+}(u) \ =\ {{\int}_{0}^{b}} G\left(u^{\prime}(t)\right)\,dt +\frac{1}{p}\|u\|_{p}^{p} -{{\int}_{0}^{b}} K_{+}\left(t,u(t)\right)\,dt \quad\forall u\in W. $$

Clearly *ψ*_+_ is coercive (see Eq. 2.3 and Eq. 3.5). Also, it is sequentially weakly lower semicontinuous. Therefore, we can find $\widetilde {u}\in W$, such that

3.6 $$ \psi_{+}(\widetilde{u}) \ =\ \inf_{u\in W}\psi_{+}(u) \ =\ \widetilde{m}_{+}. $$

Note that

$$\psi_{+}(\xi) \ =\ -{{\int}_{0}^{b}} f(t,\xi)\xi\,dt \ <\ 0 $$

(see hypothesis *H*(*f*)_1_(iii) and Eq. 3.5), so

$$\psi_{+}(\widetilde{u}) \ =\ \widetilde{m}_{+} \ <\ 0 \ =\ \psi_{+}(0), $$

hence $\widetilde {u}\ne 0$. From Eq. 3.6, we have

$$\psi_{+}^{\prime}(\widetilde{u}) \ =\ 0, $$

so

3.7 $$ A(\widetilde{u})+\left|{\vphantom{a^a}}\widetilde{u}\right|{~}^{p-2}\widetilde{u} \ =\ N_{k_{+}}(\widetilde{u}). $$

On Eq. 3.7, we act with $(\xi -\widetilde {u})^{+}\in W$. Then, using Eq. 3.5 and hypothesis *H*(*f*)_1_(iii), we have

$$\begin{array}{@{}rcl@{}} &&\left\langle A(\widetilde{u}),\ (\xi-\widetilde{u})^{+}\right\rangle +{{\int}_{0}^{b}} |\widetilde{u}|^{p-2}\widetilde{u}(\xi-\widetilde{u})^{+}\,dz\\ & = &{{\int}_{0}^{b}}\left(f(t,\xi)+\xi^{p-1}\right)(\xi-\widetilde{u})^{+}\,dz\\ & \geqslant &{{\int}_{0}^{b}} \xi^{p-1}(\xi-\widetilde{u})^{+}\,dt, \end{array} $$

so

$${\int}_{\{\xi>\widetilde{u}\}}a\left(|\widetilde{u}^{\prime}|\right)\widetilde{u}^{\prime}(-\widetilde{u})'\,dt -{\int}_{\{\xi>\widetilde{u}\}} \left(\xi^{p-1}-|\widetilde{u}|^{p-2}\widetilde{u}\right)(\xi-\widetilde{u})\,dt \ \geqslant\ 0, $$

so

$$-c_{0}\left\|\left((\xi-\widetilde{u})^{+}\right)'\right\|_{p}^{p} -{\int}_{\{\xi>\widetilde{u}\}} \left(\xi^{p-1}-|\widetilde{u}|^{p-2}\widetilde{u}\right)(\xi-\widetilde{u})\,dt \ \geqslant\ 0. $$

If $p\geqslant 2$, then $(\xi ^{p-1}-|\widetilde {u}|^{p-2}\widetilde {u})(\xi -\widetilde {u})\geqslant c_{1} |\xi -\widetilde {u}|^{p}$ for some *c*_1_>0. So

$$-c_{0}\left\|\left((\xi-\widetilde{u})^{+}\right)'\right\|_{p}^{p} -c_{1}\left\|(\xi -\widetilde{u})^{+}\right\|_{p}^{p} \ \geqslant\ 0, $$

hence $\xi \leqslant \widetilde {u}$.

If 1< *p* < 2, then

$$(\xi^{p-1}-|\widetilde{u}|^{p-2}\widetilde{u})(\xi-\widetilde{u}) \ \geqslant\ c_{2}|\xi-\widetilde{u}|^{2}\frac{1}{(1+\xi+|\widetilde{u}|)^{2-p}} \ \geqslant\ c_{3}|\xi-\widetilde{u}|^{2}, $$

for some *c*_2_,*c*_3_>0. Therefore

$$-c_{0}\big\|\left((\xi-\widetilde{u})^{+}\right)'\big\|_{p}^{p} -c_{3}\big\|(\xi-\widetilde{u})^{+}\big\|_{2}^{2} \ \geqslant\ 0, $$

hence $\xi \leqslant \widetilde {u}$.

Next on Eq. 3.7, we act with $(\widetilde {u}-w_{+})^{+}\in W$. Then, using Eq. 3.5 and hypothesis *H*(*f*)_1_(ii), we have

$$\begin{array}{@{}rcl@{}} & &\left\langle A(\widetilde{u}),\ (\widetilde{u}-w_{+})^{+}\right\rangle +{{\int}_{0}^{b}} \widetilde{u}^{p-1}(\widetilde{u}-w_{+})^{+}\,dt\\ & = &{{\int}_{0}^{b}} \left(f(t,w_{+})+w_{+}^{p-1}\right)(\widetilde{u}-w_{+})^{+}\,dt\\ & \leqslant &\left\langle A(w_{+}),\ (\widetilde{u}-w_{+})^{+}\right\rangle +{{\int}_{0}^{b}} w_{+}^{p-1}(\widetilde{u}-w_{+})^{+}\,dt, \end{array} $$

so

$$\begin{array}{@{}rcl@{}} {\int}_{\{\widetilde{u}>w_{+}\}}\left( a\left(|\widetilde{u}^{\prime}|\right)\widetilde{u}^{\prime} -a\left(|w_{+}^{\prime}|\right)w_{+}^{\prime}\right)(\widetilde{u}^{\prime}-w_{+}^{\prime})\,dt &&\\ +{\int}_{\{\widetilde{u}>w_{+}\}} \left(\widetilde{u}^{p-1}-w_{+}^{p-1}\right)(\widetilde{u}-w_{+})\,dt & \leqslant &0, \end{array} $$

so $\widetilde {u}\leqslant w_{+}$ (as before, see hypothesis *H*(*a*)(i)).

Therefore, we have proved that $\widetilde {u}\in [\xi ,w_{+}]$. This by virtue of Eq. 3.5 and Eq. 3.7 implies that

$$A(\widetilde{u})\ =\ N_{f}(\widetilde{u}), $$

so

$$\left\{ \begin{array}{l} -\left(a\left(|\widetilde{u}^{\prime}(t)|\right)\widetilde{u}^{\prime}(t)\right)'\ =\ f\left(t,\widetilde{u}(t)\right)\quad\textrm{a.e. on}\ T,\\ \widetilde{u}(0)=\widetilde{u}(b),\ \widetilde{u}^{\prime}(0)=\widetilde{u}^{\prime}(b) \end{array} \right. $$

and thus $\widetilde {u}\in \widehat {C}^{1}(T)$ is a solution of Eq. 1.1 in the order interval [*ξ*,*w*_+_]. This proves Claim 1.*Claim 2.* Problem (1.1) has the smallest solution in the order interval [*ξ*,*w*_+_].

Let $\mathcal {Y}_{+}$ be the set of solutions of problem (1.1) in the order interval [*ξ*,*w*_+_]. From Claim 1, we know that $\mathcal {Y}_{+}\ne \emptyset $. Let $C\subseteq \mathcal {Y}_{+}$ be a chain (i.e. a nonempty totally ordered subset of $\mathcal {Y}_{+}$). From Dunford-Schwartz [10, p.336], we know that we can find a sequence $\{u_{n}\}_{n\geqslant 1}\subseteq C$, such that

$$\inf C \ =\ \inf_{n\geqslant 1}u_{n}. $$

We have

3.8 $$ A(u_{n}) \ =\ N_{f}(u_{n}) \quad\text{and}\quad u_{n}\ \in\ [\xi,w_{+}] \quad\forall n\geqslant 1, $$

so the sequence $\{u_{n}\}_{n\geqslant 1}\subseteq W$ is bounded.

So, we may assume that

3.9 $$ u_{n}\ \stackrel{w}{\longrightarrow}\ u\ \text{in}\ W\quad\text{and}\quad u_{n}\ \longrightarrow\ u\ \text{in}\ C(T). $$

Acting on Eq. 3.8 with *u*_*n*_−*u*∈*W*, passing to the limit as $n\rightarrow +\infty $ and using Eq. 3.9, we obtain

$$\lim_{n\rightarrow +\infty} \left\langle A(u_{n}),\ u_{n}-u\right\rangle \ =\ 0, $$

so

3.10 $$ u_{n}\ \longrightarrow\ u\ \text{in}\ W $$

(see Proposition 2.7), with *u*∈[*ξ*,*w*_+_].

So, if in Eq. 3.8, we pass to the limit as $n\rightarrow +\infty $ and use Eq. 3.10, we have

$$A(u)\ =\ N_{f}(u),\quad u\in [\xi,w_{+}], $$

so

$$u\in\mathcal{Y}_{+}\quad\text{and}\quad u=\inf C. $$

Since *C* is an arbitrary chain, from the Kuratowski-Zorn lemma, we infer that $\mathcal {Y}_{+}$ has a minimal element $\widehat {u}\in \mathcal {Y}_{+}$. Exploiting the monotonicity of *A* (see Proposition 2.3), as in Aizicovici-Papageorgiou-Staicu [3] (see Lemma 1 and Proposition 8), we show that $\mathcal {Y}_{+}$ is downward directed (i.e. if $u_{1},u_{2}\in \mathcal {Y}_{+}$, then we can find $u\in \mathcal {Y}_{+}$, such that $u\leqslant u_{1}$, $u\leqslant u_{2}$). Hence, $\widehat {u}\in \mathcal {Y}_{+}$ is the smallest solution of Eq. 1.1 in the order interval [*ξ*,*w*_+_]. This proves Claim 2.

Now suppose that $\{\xi _{n}\}_{n\geqslant 1}\subseteq (0,\delta _{0}]$ is a sequence, such that *ξ*_*n*_↘0. By virtue of Claim 2, for every $n\geqslant 1$, we can find the smallest solution $u_{n}\in \widehat {C}^{1}(T)$ of Eq. 1.1 in [*ξ*_*n*_,*w*_+_]. Then, $\{u_{n}\}_{n\geqslant 1}\subseteq W$ is bounded decreasing, and we may assume that

$$u_{n}\ \stackrel{w}{\longrightarrow}\ u_{*}\ \text{in}\ W\quad\text{and}\quad u_{n}\ \longrightarrow\ u_{*}\ \text{in}\ C(T), $$

so $\|u_{*}\|_{\infty }\geqslant \delta _{0}$ (see Proposition 3.2) and thus *u*_∗_≠0.

Also as above, via Eq. 3.8 and Proposition 3.9, we have

$$A(u_{*})~=~N_{f}(u_{*}), $$

hence $u_{*}\in \widehat {C}_{+}\setminus \{0\}$ is a solution of Eq. 1.1. Moreover, hypothesis *H*(*f*)_1_(iv) and the nonlinear maximum principle of Pucci-Serrin [22, p. 120] imply that $u_{*}\in \text {int}\, \widehat {C}_{+}$.

Similarly, for the negative solution, we choose *ξ*∈[−*δ*_0_,0) and consider the order interval

$$[w_{-},\xi] \ =\ \left\{ u\in W:\ w_{-}(t)\leqslant u(t)\leqslant\xi\ \textrm{for almost all}\ t\in T \right\}. $$

Then, the set $\mathcal {Y}_{-}$ of nontrivial solutions of problem (1.1) in [*w*_−_,*ξ*] is nonempty and upward directed (i.e. if $v_{1},v_{2}\in \mathcal {Y}_{-}$, then we can find $v\in \mathcal {Y}_{-}$, such that $v_{1}\leqslant v$, $v_{2}\leqslant v$; see Aizicovici-Papageorgiou-Staicu [3]). So, as above, we can find the biggest nontrivial negative solution $v_{*}\in -\text {int}\,\widehat {C}_{+}$ of problem (1.1). □

Using these extremal nontrivial constant sign solutions, we will produce a nodal (sign changing) solution. To this end, we need to restrict further the behaviour of *f*(*t*,⋅) near zero. More precisely, the new hypotheses on the reaction *f* are the following: *H(f)*_2_ $f\colon T\times \mathbb {R}\longrightarrow \mathbb {R}$ is a Carathéodory function, such that *f*(*t*,0)=0 for almost all *t*∈*T*, hypotheses *H*(*f*)_2_(i), (ii) and (iv) are the same as the corresponding hypotheses *H*(*f*)_1_(i), (ii), (iv) and

(iii) there exist *q*∈(1,*τ*) and *δ*_0_>0, such that

$$q F(t,\zeta)\ \geqslant\ f(t,\zeta)\zeta\ >\ 0 \quad\textrm{for almost all}\ t\in T,\ \text{all}\ 0<|\zeta|\leqslant\delta_{0}, $$

and

$$\text{ess} \inf\limits_{{}T} F(\cdot,\delta_{0})\ >\ 0. $$

### *Remark 3.5*

Clearly hypothesis *H*(*f*)_2_(iii) is more restrictive than hypothesis *H*(*f*)_1_(iii) and we can easily see that it implies that

$$F(t,\zeta)\ \geqslant\ c_{3}|\zeta|^{q} \quad\textrm{for almost all}\ t\in T, \ \text{all}\ |\zeta|\leqslant\delta_{0} $$

with some *c*_3_>0.

With these stronger hypotheses on *f*(*t*,⋅), we can produce a nodal solution.

### **Proposition 3.6**

*If hypotheses H(a) and H(f)*_2_ *hold, then problem (* 1.1 *) has a nodal solution* $y_{0}\in \widehat {C}^{1}(T)$.

### *Proof*

Let $u_{*}\in \text {int}\,\widehat {C}_{+}$ and $v_{*}\in \text {int}\,\widehat {C}_{+}$ be the two extremal nontrivial constant sign solutions produced in Proposition 3.4. Using them, we introduce the following truncation-perturbation of the reaction *f*(*t*,⋅):

3.11 $$ \beta(t,\zeta) \ =\ \left\{ \begin{array}{lll} f(t,v_{*}(t))+|v_{*}(t)|^{p-2}v_{*}(t) & \text{if} & \zeta<v_{*}(t),\\ f(t,\zeta)+|\zeta|^{p-2}\zeta & \text{if} & v_{*}(t)\leqslant\zeta\leqslant u_{*}(t),\\ f(t,u_{*}(t))+u_{*}(t)^{p-1} & \text{if} & u_{*}<\zeta. \end{array} \right. $$

This is a Carathéodory function. We set

$$B(t,\zeta) \ =\ {\int}_{0}^{\zeta}\beta(t,s)\,ds $$

and consider the *C* ^1^-functional $\sigma \colon W\longrightarrow \mathbb {R}$, defined by

$$\sigma(u) \ =\ {{\int}_{0}^{b}}G\left(u^{\prime}(t)\right)\,dt+\frac{1}{p}\|u\|_{p}^{p} -{{\int}_{0}^{b}} B\left(t,u(t)\right)\,dt \quad\forall u\in W. $$

Also, let

$$\begin{array}{@{}rcl@{}} \beta_{\pm}(t,\zeta) & = & \beta\left(t,\pm\zeta^{\pm}\right),\\ B_{\pm}(t,\zeta) & = & {\int}_{0}^{\zeta}\beta_{\pm}(t,s)\,ds \end{array} $$

and consider the *C* ^1^-functional $\sigma _{\pm }\colon W\longrightarrow \mathbb {R}$, defined by

$$\sigma_{\pm}(u) \ =\ {{\int}_{0}^{b}} G\left(u^{\prime}(t)\right)\,dt +\frac{1}{p}\|u\|_{p}^{p} -{{\int}_{0}^{b}} B_{\pm}\left(t,u(t)\right)\,dt \quad\forall u\in W. $$

As in the proof of Proposition 3.4, we can show that

$$K_{\sigma}\ \subseteq\ [v_{*},u_{*}],\quad K_{\sigma_{+}}\ \subseteq\ [0,u_{*}],\quad K_{\sigma_{-}}\ \subseteq\ [v_{*},0]. $$

The extremality of the solutions *u*_∗_ and *v*_∗_ implies that

3.12 $$ K_{\sigma}\ \subseteq\ [v_{*},u_{*}],\quad K_{\sigma_{+}}\ =\ \{0,u_{*}\},\quad K_{\sigma_{-}}\ =\ \{v_{*},0\}. $$

*Claim.* *u*_∗_ and *v*_∗_ are local minimizers of *σ*.

Evidently, the functional *σ*_+_ is coercive (see Eq. 3.11). Also, it is sequentially weakly lower semicontinuous. So, we can find $\widehat {u}\in W$, such that

$$\sigma_{+}(\widehat{u}) \ =\ \inf_{u\in W}\sigma_{+}(u). $$

As before, hypothesis *H*(*f*)_2_ (iii) implies that

$$\sigma_{+}(\widehat{u})\ <\ 0\ =\ \sigma_{+}(0), $$

hence $\widehat {u}\ne 0$. Since $\widehat {u}\in K_{\sigma _{+}}$, from Eq. 3.12, it follows that $\widehat {u}=u_{*}\in \text {int}\,\widehat {C}_{+}$. But note that

$$\sigma\big|_{\widehat{C}_{+}} \ =\ \sigma_{+}\big|_{\widehat{C}_{+}}. $$

Because $u_{*}\in \text {int}\,\widehat {C}_{+}$, it follows that *u*_∗_ is a local $\widehat {C}^{1}(T)$-minimizer of *σ*. Invoking Proposition 2.8, we infer that *u*_∗_ is a local *W*-minimizer of *σ*.

Similarly for *v*_∗_ using this time the functional *σ*_−_. This proves the Claim.

Without any loss of generality, we may assume that $\sigma (v_{*})\leqslant \sigma (u_{*})$ (the analysis is similar if the opposite inequality holds). Then, as in Aizicovici-Papageorgiou-Staicu [2, Proposition 29] or Gasiński-Papageorgiou [14, proof of Theorem 3.4], we can find *ϱ*∈(0,1) small, such that

3.13 $$ \sigma(v_{*})\ \leqslant\ \sigma(u_{*}) \ <\ \inf\left\{\sigma(u):\ \|u-u_{*}\|=\varrho\right\} \ =\ \eta_{\varrho},\quad \|v_{*}-u_{*}\|\ >\ \varrho. $$

Since the functional *σ* is coercive (see Eq. 3.11), it satisfies the Palais-Smale condition. Indeed, let $\{u_{n}\}_{n\geqslant 1}\subseteq W$ be such that

3.14 $$ \textrm{the squence}\ \left\{\sigma(u_{n})\right\}_{n\geqslant 1}\subseteq\mathbb{R}\ \textrm{is bounded and}\ \sigma^{\prime}(u_{n})\longrightarrow 0\ \text{in}\ W^{*}. $$

From the coercivity of *σ*, it follows that $\{u_{n}\}_{n\geqslant 1}\subseteq W$ is bounded, and so we may assume that

$$u_{n}\ \stackrel{w}{\longrightarrow}\ u\ \text{in}\ W\quad\text{and}\quad u_{n}\ \longrightarrow\ u\ \text{in}\ C(T). $$

Then as before, using the convergence in Eq. 3.14 and Proposition 2.7, we conclude that

$$u_{n}\ \longrightarrow\ u\quad\text{in}\ W, $$

hence *σ* satisfies the Palais-Smale condition. This fact and (3.13) permit the use of the mountain pass theorem (see Theorem 2.1). So, we can find *y*_0_∈*W*, such that

$$y_{0}\in K_{\sigma}\quad\text{and}\quad \eta_{\varrho}\leqslant\sigma(y_{0}), $$

so $y_{0}\in \widehat {C}^{1}(T)$ solves problem (1.1), *y*_0_∈[*v*_∗_,*u*_∗_] (see Eq. 3.12), *y*_0_≠*v*_∗_ and *y*_0_≠*u*_∗_ (see Eq. 3.13).

It remains to show that *y*_0_ is nontrivial. We know that *y*_0_ is a critical point of *σ* of mountain pass type, while hypothesis *H*(*f*)_2_(iii) implies the presence of a concave term near the origin. Hence, the origin is a critical point of a different kind and must be different from *y*_0_. An easy way to establish this rigorously is to use critical groups. Since *y*_0_∈*K*_*σ*_ is of mountain pass type, we have

3.15 $$ C_{1}(\sigma,y_{0})\ \ne\ 0 $$

(see Chang [7, p. 89]). On the other hand, hypothesis *H*(*f*)_2_(iii) and Proposition 2.1 of Moroz [20] imply that

3.16 $$ C_{k}(\sigma,0)\ =\ 0 \quad\forall k\geqslant 0. $$

Comparing Eqs. 3.15 and 3.16, we infer that *y*_0_≠0. Therefore,

$$y_{0}\ \in\ [v_{*},u_{*}],\quad y_{0}\not\in \{0,u_{*},v_{*}\}. $$

The extremality of *u*_∗_,*v*_∗_ implies that $y_{0}\in \widehat {C}^{1}(T)$ is a nodal solution of Eq. 1.1. □

So, we can now state the first multiplicity theorem for problem (1.1).

### **Theorem 3.7**

*If hypotheses H(a) and H(f)*_2_ *hold, then problem (* 1.1 *) has at least three nontrivial solutions*

$$u_{0}\ \in\ \text{int}\,\widehat{C}_{+},\quad v_{0}\ \in\ -\text{int}\,\widehat{C}_{+},\quad\text{and}\quad y_{0}\ \in\ \widehat{C}^{1}(T)\ \text{nodal}. $$

As we already mentioned, hypothesis *H*(*f*)_2_(iii) implies that the reaction *f*(*t*,⋅) near *ζ*=0 exhibits a “concave” term. We can relax this restriction and allow nonlinearities with more general growth near *ζ*=0, provided that we restrict the growth of *ζ*↦*a*(*ζ*).

So, the new hypotheses on the functions *a* and *f* are the following: *H(a)*′ *a* : (0, + ∞) → (0, + ∞) is a *C* ^1^-function, such that hypotheses *H*(*a*)^′^(i), (ii) and (iv) are the same as the corresponding hypotheses *H*(*a*)(i), (ii), (iv) and (iii) there exists *c*_1_>0 such that

$$\big| a(|\zeta|)\zeta\big|\ \leqslant\ c_{1} |\zeta|^{p-1} \quad\forall \zeta\in\mathbb{R}. $$

### *Remark 3.8*

The more restrictive growth imposed in *H*(*a*)^′^(iii) excludes from consideration the scalar (*p*,*q*)-Laplacian and the scalar *p*-generalized mean curvature differential operator. On the other hand, it applies to the scalar *p*-Laplacian corresponding to *a*(|*ζ*|)*ζ*=|*ζ*|^*p*−2^ *ζ* with $1<p<+\infty $. Other possibilities are:

$$\begin{array}{@{}rcl@{}} a(|\zeta|)\zeta & = & |\zeta|^{p-2}\zeta+\frac{|\zeta|^{p-2}\zeta}{1+|\zeta|^{p}},\\ a(|\zeta|)\zeta & = & |\zeta|^{p-2}\zeta+\ln\left(1+|\zeta|^{p-2}\right)\zeta,\\ a(|\zeta|)\zeta & = &\left\{ \begin{array}{lll} |\zeta|^{p-2}\zeta+|\zeta|^{r-2}\zeta & \text{if} & |\zeta|\leqslant 1,\\ 2|\zeta|^{p-2}\zeta+|\zeta|^{\tau-2}\zeta & \text{if} & |\zeta|> 1, \end{array} \right. \end{array} $$

where $1<\tau <p<r<+\infty $, *r*=*p*+*τ*−1.

Note that this new growth condition on *σ* implies that

$$\frac{c_{0}}{p}|\zeta|^{p}\ \leqslant\ G(\zeta)\ \leqslant\ \frac{c_{1}}{p}|\zeta|^{p} \quad\forall \zeta\in\mathbb{R}. $$

*H(f*)_3_ *f* : *T* × ℝ → ℝ is a Carathéodory function, such that *f*(*t*,0)=0 for almost all *t*∈*T*, hypotheses *H*(*f*)_3_(i), (ii), (iii) and (iv) are the same as the corresponding hypotheses *H*(*f*)_1_(i), (ii), (iii) and (iv) and in addition (v) there exist $\widehat {\delta }_{0}>0$ and *𝜗*>*λ*_1_, such that

$$\frac{c_{1}\vartheta|\zeta|^{p}}{p}\ \leqslant\ F(t,\zeta) \quad\textrm{for almost all}\ t\in T,\ \text{all}\ |\zeta|\leqslant\widehat{\delta}_{0}, $$

with *c*_1_>0 as in hypothesis *H*(*a*)^′^(iii).

### *Remark 3.9*

Evidently, hypothesis *H*(*f*)_3_(*v*) permits reactions *f*(*t*,*ζ*) which are (*p*−1)-linear near zero, a case which was excluded by hypothesis *H*(*f*)_2_(iii).

The previous analysis concerning nontrivial solutions of constant sign remains valid. What changes is the proof of the existence of a nodal solution.

### **Proposition 3.10**

*If hypotheses H(a)* ^′^ *and H(f)*_3_ *hold, then problem (* 1.1 *) has a nodal solution* $y_{0}\in \widehat {C}^{1}(T)$.

### *Proof*

As before (see the proof of Proposition 3.6), using the extremal nontrivial constant sign solutions $u_{*}\in \text {int}\,\widehat {C}_{+}$ and $v_{*}\in -\text {int}\,\widehat {C}_{+}$, truncating *f*(*t*,⋅) at $\left \{u_{*}(t),v_{*}(t)\right \}$ (see Eq. 3.11) and employing the mountain pass theorem (see Theorem 2.1), we obtain a solution $y_{0}\in \widehat {C}^{1}(T)$ of problem (1.1), such that *y*_0_∈[*v*_∗_,*u*_∗_], *y*_0_∉{*u*_∗_,*v*_∗_} and

3.17 $$ \sigma(y_{0}) \ =\ \inf_{\gamma\in{\Gamma}}\max_{0\leqslant t\leqslant 1}\sigma\left(\gamma(t)\right), $$

with ${\Gamma }=\left \{\gamma \in C\left ([0,1];W\right ):\ \gamma (0)=v_{*},\ \gamma (1)=u_{*}\right \}$. We need to show that *y*_0_≠0 and then due to extremality of *u*_∗_ and *v*_∗_, we will have that $y_{0}\in \widehat {C}^{1}(T)$ is nodal. To show the nontriviality of *y*_0_, we will use the minimax expression in Eq. 3.17. According to this characterization of *φ*(*y*_0_), it suffices to produce a path *γ*_∗_∈Γ, such that $\sigma \big |_{\gamma _{*}}<0$.

To this end, let

$$M\ =\ W\cap\partial B_{1}^{L^{p}}\quad\text{and}\quad M_{c}\ =\ M\cap \widehat{C}^{1}(T). $$

We endow *M* with the relative *W*-topology and *M*_*c*_ with the relative $\widehat {C}^{1}(T)$-topology.

Evidently, *M*_*c*_ is dense in *M* and $C\left ([-1,1];M_{c}\right )$ is dense in $C\left ([-1,1];M\right )$. We consider the following sets of points

$$\begin{array}{@{}rcl@{}} \widehat{\Gamma} & = &\left\{\widehat{\gamma}\in C\left([-1,1];M\right):\ \widehat{\gamma}(-1)=-\widehat{u}_{0},\ \widehat{\gamma}(1)=\widehat{u}_{0}\right\},\\ \widehat{\Gamma}_{c} & = &\left\{\widehat{\gamma}\in C\left([-1,1];M_{c}\right):\ \widehat{\gamma}(-1)=-\widehat{u}_{0},\ \widehat{\gamma}(1)=\widehat{u}_{0}\right\}. \end{array} $$

Then $\widehat {\Gamma }_{c}$ is dense in $\widehat {\Gamma }$ and so by virtue of Proposition 2.4, we can find $\widehat {\gamma }_{0}\in \widehat {\Gamma }_{c}$, such that

3.18 $$ \max_{-1\leqslant s\leqslant 1}\left\|\frac{d}{dt}\widehat{\gamma}_{0}(s)\right\|_{p}^{p} \ <\ \vartheta. $$

Since $\widehat {\gamma }_{0}\in \widehat {\Gamma }_{c}$ and $v_{*}\in -\text {int}\,\widehat {C}_{+}$, $u_{*}\in \text {int}\,\widehat {C}_{+}$, we can find *ε*>0 small, such that

3.19 $$ v_{*}(t)\leqslant\varepsilon\widehat{\gamma}(s)(t)\leqslant u_{*}(t),\quad \varepsilon\big|\widehat{\gamma}_{0}(s)(t)\big|\leqslant\widehat{\delta}_{0} \quad\forall s\in [-1,1],\ t\in T. $$

Then, assuming without any loss of generality that $\widehat {\delta }_{0}\leqslant \min \{-c_{-},c_{+}\}$, we have

3.20 $$\begin{array}{@{}rcl@{}} \sigma\left(\varepsilon\widehat{\gamma}_{0}(s)\right) & = &{{\int}_{0}^{b}} G\left(\frac{d}{dt}\varepsilon\widehat{\gamma}_{0}(s)(t)\right)dt -{{\int}_{0}^{b}} B\left(t,\varepsilon\widehat{\gamma}_{0}(s)(t)\right)dt\cr & \leqslant &\frac{c_{1}\varepsilon^{p}}{p}\bigg\|\frac{d}{dt}\widehat{\gamma}_{0}(s)\bigg\|_{p}^{p} -\frac{c_{1}}{p}\varepsilon^{p}\vartheta \ <\ \frac{c_{1}\varepsilon^{p}}{p}(\vartheta-\vartheta) \ =\ 0 \end{array} $$

(see Eqs. 3.11, 3.18, 3.19 and hypothesis *H*(*f*)_3_(*v*) and recall that $\left \|\widehat {\gamma }_{0}(s)\right \|_{p}=1$ for all *s*∈[−1,1]).

Therefore, if $\gamma _{0}=\varepsilon \widehat {\gamma }_{0}$, then *γ*_0_ is a continuous path in *W* which connects $-\varepsilon \widehat {u}_{0}$ and $\varepsilon \widehat {u}_{0}$, and we have

3.21 $$ \sigma\big|_{\gamma_{0}}\ <\ 0 $$

(see Eq. 3.20).

Next, we produce a continuous path in *W*, which connects $\varepsilon \widehat {u}_{0}$ and *u*_∗_ and along which *σ* is strictly negative.

Let

$$a \ =\ \inf_{W}\sigma_{+}\ <\ 0\ =\ \sigma_{+}(0) $$

(see the proof of Proposition 3.6). Recall *σ*_+_ being coercive, it satisfies the Palais-Smale condition. So, we can apply the second deformation theorem (see Theorem 2.2) and obtain a deformation $h\colon [0,1]\times \left (\sigma _{+}^{0}\setminus K_{\sigma _{+}}^{0}\right )\longrightarrow \sigma _{+}^{0}$, such that

$$h(t,\cdot)\left|{~}_{K_{\sigma_{+}}^{a}}\ =\ id\right|{~}_{K_{\sigma_{+}}^{a}} $$

and

3.22 $$ h\left(1,\sigma_{+}^{0}\setminus K_{\sigma_{+}}^{0}\right)\ \subseteq\ \sigma_{+}^{a}\ =\ \{u_{*}\}, $$

3.23 $$ \sigma\left(h(\tau,\zeta)\right)\ \leqslant\ \sigma_{+}\left(h(s,\zeta)\right) \quad\forall \tau,s\in [0,1],\ s\leqslant\tau,\ \zeta\in\sigma_{+}^{0}\setminus\{0\}. $$

Let

$$\gamma_{+}(s) \ =\ h\left(s,\varepsilon\widehat{u}_{0}\right)^{+} \quad\forall s\in [0,1] $$

(see Eq. 3.21). Then

$$\begin{array}{@{}rcl@{}} \gamma_{+}(0) & = & h(0,\varepsilon\widehat{u}_{0})^{+}\ =\ \varepsilon\widehat{u}_{0}^{+}\ =\ \varepsilon\widehat{u}_{0},\\ \gamma_{+}(1) & = & h(1,\varepsilon\widehat{u}_{0})^{+}\ =\ u_{*}^{+}\ =\ u_{*} \end{array} $$

(see Eq. 3.22).

Hence, *γ*_+_ is a continuous path in *W* which connects $\varepsilon \widehat {u}_{0}$ and *u*_∗_. Also, from Eqs. 3.21 and 3.23, we have $\sigma _{+}\left |{\vphantom {1^1}}\right ._{\gamma _{+}}<0$. If

$$W_{+} \ =\ \left\{u\in W:\ u(t)\geqslant 0\ \textrm{for all}\ t\in T\right\}, $$

then

$$\sigma_{+}\left|{~}_{W_{+}}\ =\ \sigma\right|{~}_{W_{+}}. $$

Also $\text {range}\,\gamma _{+}\subseteq W_{+}$. Therefore

3.24 $$ \sigma\left|{\vphantom{a^a}}\right._{\gamma_{+}}\ <\ 0. $$

In a similar fashion, we produce a continuous path *γ*_−_ in *W* which connects $-\varepsilon \widehat {u}_{0}$ and *v*_∗_ and such that

3.25 $$ \sigma\left|{\vphantom{a^a}}\right._{\gamma_{-}}\ <\ 0. $$

We concatenate *γ*_−_,*γ*_0_,*γ*_+_ and produce *γ*_∗_∈Γ, such that

$$\sigma\left|{\vphantom{a^a}}\right._{\gamma_{*}}\ <\ 0 $$

(see Eqs. 3.21, 3.24 and 3.25), so

$$\sigma(y_{0})\ <\ 0\ =\ \sigma(0) $$

(see Eq. 3.17) and thus $y_{0}\in \widehat {C}^{1}(T)$ is a nodal solution of Eq. 1.1. □

So, we can now state the second multiplicity theorem for problem (1.1).

### **Theorem 3.11**

*If hypotheses H(a)* ^′^ *and H(f)*_3_ *hold, then problem (* 1.1 *) has at least three nontrivial solutions*

$$u_{0}\ \in\ \text{int}\,\widehat{C}_{+},\quad v_{0}\ \in\ -\text{int}\,\widehat{C}_{+},\quad\text{and}\quad y_{0}\ \in\ \widehat{C}^{1}(T)\ \text{nodal}. $$
